# Progress in Surgery

**Published:** 1900-06-23

**Authors:** 


### PROGRESS IN SURGERY.

### GENERAL SURGERY.

**Antiseptics.**—M. Sedan[1] has introduced a new antiseptic termed aniodol, which is essentially a solution of trimethanol, and is a powerful germicide. It is employed for disinfecting instruments, the hands of the operator, wounds, and dressings. M. Pinard also states that there are no drawbacks to its use, especially in maternity practice. Powdered antiseptics are at present commonly used in the antiseptic treatment of wounds; but iodoform, which has been employed for many years, is still much discredited, and substitutes for the same are anxiously looked out for, partly because it is so highly toxic to the system and too frequently causes poisoning, and partly because of its strong and sometimes disagreeable odour. Airol, the bismuth combination, containing two negative constituents, iodine and gallic acid, is one of these substitutes. Julian Marcuse[2] gives a *résumé* of this antiseptic, which clearly illustrates its advantages and disadvantages. He believes that it has proved to be of value as a substitute for iodoform in all external injuries and wounds in which it is essential to employ a powdered antiseptic which limits secretion, assists the granulation process, and effects rapid healing over of the skin. In comparison with iodoform, it has in all such cases the great advantage of being non-irritating and odourless. Most excellent results are obtained in ulcers of the leg, diseases of the skin and genitals, hard and soft chancre, buboes after being opened, and in eczema-intertrigo. The employment and value of a 10 per cent. airol emulsion (airol 2·0, glycerine and aqua distill. āā 10·0) in gonorrhœa, chronic otorrhœa, and tuberculous laryngeal ulcers is also recommended. Schleich[3] disputes the assertion that bacteria are the chief source of infection. He looks upon them as the accompaniments of uncleanliness, of which their presence is sufficient proof, but he rejects the idea that they are the cause of sepsis and pyæmia, for the reason that these diseases occur in the presence of very different forms of bacteria. He insists that the operator should disinfect himself immediately after contact with infectious material, so that at any time he may be looked upon as nearly aseptic. Hand brushes he describes as universal labyrinths for grease and dirt. He also discards all sorts of chemical methods of purification, partly because they do not destroy the bacteria, and partly because they cannot be thoroughly carried out. He advises a mechanical washing away of the bacteria, and therefore uses a "marble soap," which is made of a special alkali (steral), and contains marble dust, to take the place of brushes. After the hands are washed they are smeared with wax paste to close the ducts of the sweat and fat glands, which no method is capable of disinfecting. Schleich rejects all drainage, and uses silk instead of catgut, as he does not believe that the latter can be perfectly sterilised. He boils his silk, and immerses it in gelatine to prevent it from infection; the gelatine is melted off with hot water at the time of use. The method of disinfection of the hands of the surgeon, &c., by a solution of biniodide of mercury and methylated spirit (1 in 500) is now employed by nearly all Mr. Lockwood's[4] surgical colleagues at the

Great Northern and St. Bartholomew's Hospitals. As regards the hands, after the nails have been cut close and all outside dirt and grease removed, the biniodide solution which is also used for the patient's skin is employed. Not less than half a pint of this fluid should be placed in a capacious bowl ready at hand. The results of the septic testing of the hands, skin, &c., have made steady progress of late in Mr. Lockwood's wards. At the recent German Surgical Congress, Dr. Sarwey[5] described a large series of experiments which he had made in disinfection of the hands by different methods. He considered that the best method of bacteriological examination was to rub a sterilised piece of hard wood on the hands, previously moistened with sterilised water. Washing and brushing the hands, even for hours, with ordinary soap and water did not diminish the number of microbes upon the skin to any considerable degree. He thought there was no method which rendered the hands absolutely free from microbes, but the method of Mikulicz with spirit gave relatively the best results, diminishing the number of microbes considerably. The same was true of Ahlfield's method of disinfecting with hot water and alcohol. Freeman,[6] while admitting that much may be accomplished by sweating the hands in a hot-air oven, by immersing the gloved hands in hot water, or by prolonged soaking in hot water, yet thinks that at present the only reliable means of rendering the hands aseptic is to encase them in intact sterilised rubber gloves; cotton gloves, although they soon become contaminated by exudations from the skin, may do some good, especially if often changed, by filtering out the bacteria, and by preventing their entrance into the wounds. Schloffer[7] reports favourably on the use in Wölfler's clinic of thin leather gloves, carefully disinfected at first by hot water, and afterwards by prolonged immersion in a 5 per cent. solution of lysol. The wearing of gloves, he says, should not lead to any carelessness in disinfecting the skin of the hands, as it may be necessary in the course of an operation to work with the bare hands.

[1] Les Noveaux Remédes, April 22, 1900, p. 187. [2] Therapist, Sept. 15, 1899, p. 231. [3] Ibid., Feb. 15, 1900, p. 53. [4] Brit. Med. Jour., Feb. 24, 1900, p. 429. [5] Brit. Med. Jour., May 5, 1900, p. 1115. [6] Epit. Brit. Med. Jour., Nov. 11, 1899. [7] Epit. Brit. Med. Jour., Mar. 7, 1900; and Archiv. f. Klin. Chir., Bd. lix., Heft. 1.

